# Effect of garlic on blood pressure: A systematic review and meta-analysis

**DOI:** 10.1186/1471-2261-8-13

**Published:** 2008-06-16

**Authors:** Karin Ried, Oliver R Frank, Nigel P Stocks, Peter Fakler, Thomas Sullivan

**Affiliations:** 1 Discipline of General Practice, The University of Adelaide, Adelaide, South Australia; 2 Discipline of Public Health, The University of Adelaide, Adelaide, South Australia

## Abstract

**Background:**

Non-pharmacological treatment options for hypertension have the potential to reduce the risk of cardiovascular disease at a population level. Animal studies have suggested that garlic reduces blood pressure, but primary studies in humans and non-systematic reviews have reported mixed results. With interest in complementary medicine for hypertension increasing, it is timely to update a systematic review and meta-analysis from 1994 of studies investigating the effect of garlic preparations on blood pressure.

**Methods:**

We searched the Medline and Embase databases for studies published between 1955 and October 2007. Randomised controlled trials with true placebo groups, using garlic-only preparations, and reporting mean systolic and/or diastolic blood pressure (SBP/DBP) and standard deviations were included in the meta-analysis. We also conducted subgroup meta-analysis by baseline blood pressure (hypertensive/normotensive), for the first time. Meta-regression analysis was performed to test the associations between blood pressure outcomes and duration of treatment, dosage, and blood pressure at start of treatment.

**Results:**

Eleven of 25 studies included in the systematic review were suitable for meta-analysis. Meta-analysis of all studies showed a mean decrease of 4.6 ± 2.8 mm Hg for SBP in the garlic group compared to placebo (n = 10; p = 0.001), while the mean decrease in the hypertensive subgroup was 8.4 ± 2.8 mm Hg for SBP (n = 4; p < 0.001), and 7.3 ± 1.5 mm Hg for DBP (n = 3; p < 0.001). Regression analysis revealed a significant association between blood pressure at the start of the intervention and the level of blood pressure reduction (SBP: R = 0.057; p = 0.03; DBP: R = -0.315; p = 0.02).

**Conclusion:**

Our meta-analysis suggests that garlic preparations are superior to placebo in reducing blood pressure in individuals with hypertension.

## Background

Hypertension (systolic blood pressure (SBP) ≥ 140 mm Hg; diastolic blood pressure (DBP) ≥ 90 mm Hg) is a known risk factor for cardiovascular morbidity and mortality, affecting an estimated 1 billion individuals worldwide [1]. Recently updated guidelines for the treatment of high blood pressure stress the importance of preventive strategies, and recommend extending the management of blood pressure to include pre-hypertensive individuals (SBP 120–139/DBP 80–89 mm Hg) [1]. Primary management should include relevant lifestyle modifications such as increased exercise, weight loss and dietary changes which could incorporate dietary supplementation.

Garlic (*Allium sativum*) has played an important dietary as well as medicinal role in human history [2]. Blood pressure reducing properties of garlic have been linked to its hydrogen sulphide production [3] and allicin content – liberated from alliin and the enzyme alliinase [4,5] – which has angiotensin II inhibiting and vasodilating effects, as shown in animal and human cell studies [3,6-10].

Primary studies in humans and reviews of garlic preparations and blood pressure have been inconclusive [11-40]. A meta-analysis published in 1994 reported promising results in subjects with mild hypertension but found insufficient evidence to recommend garlic for clinical therapy [41]. The increasing use of alternative and complementary therapies for hypertension [42,43] makes it timely to provide an updated systematic review and meta-analysis of trials investigating the effect of garlic preparations on blood pressure. Inclusion of additional data from studies published since 1994 has enabled subgroup meta-analyses of hypertensive and normotensive subjects.

## Methods

### Literature search

We searched the Medline, Embase and Cochrane databases for studies published between 1955 and Oct 2007 using the search terms [garlic AND ("blood pressure" OR hypertens* OR pre-hypertens* OR prehypertens*)] to identify intervention studies investigating the effect of garlic on blood pressure. We also checked reference lists of previously published systematic reviews and meta-analyses for additional primary studies [36,41].

### Study selection

**Figure 1:**
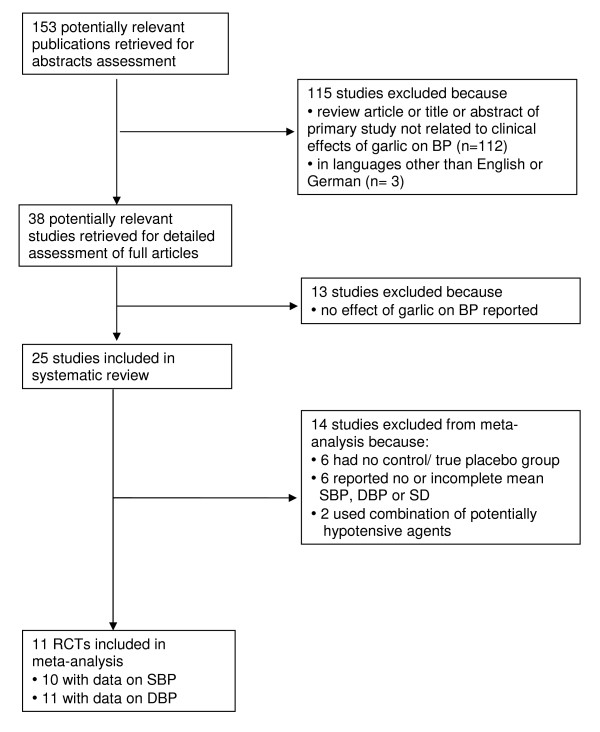
**Flow diagram of study selection for systematic review and meta-analysis**. *Abbreviations: BP, blood pressure; SBP, systolic blood pressure; DBP, diastolic blood pressure; SD, standard deviation; RCT, randomised controlled trial*

In the systematic review we included published intervention studies (these included randomised controlled trials and non-placebo controlled trials), reporting effects of garlic on blood pressure and published in English or German (Table 1 and Additional File 1). Stricter criteria were required for inclusion in meta-analysis: Only studies with placebo control groups, using garlic-only supplements, and reporting mean systolic and/or diastolic blood pressure (SBP/DBP) and standard deviation (SD) were eligible for meta-analysis (Table 1). We contacted authors of studies with suitable study design but incomplete published data (mean SBP/DBP or SD) to retrieve complete data sets for meta-analysis. Figure 1 summarises the study selection process.

**Table 1:** Characteristics of randomised controlled trials included in meta-analysis examining the effect of garlic on blood pressure

**Source**	**Study design; Intervention/control groups**	**Type of garlic preparation, Dosage, Duration**	**Number of participants in intervention vs control group**	**Mean SBP (SD) at start/end of intervention vs control in mm Hg**	**Mean DBP (SD) at start/end of intervention vs control in mm Hg**
Kandziora J 1988 (Study 1), [11]	Parallel,	Garlic powder (Kwai),	20/20	Garlic: 174 (4)/158 (10)	Garlic: 99 (3)/83 (4)
	Diuretic drug (Dytide H) + garlic/drug only	600 mg/d,	(standing)	Control: 175 (8)/169 (6)	Control: 98 (5)/90 (3)
		12 wks			
Auer et al. 1990, [12]	Parallel,	Kwai,	24/23	Garlic: 171 (21.6)/152 (19.6)	Garlic: 102 (13)/89 (4.4)
	Garlic/placebo	600 mg/d,		Control: 161 (19)/153 (19)	Control: 97 (12.9)/93 (10.6)
		12 wks			
Vorberg & Schneider 1990, [13]	Parallel,	Kwai,	20/20	Garlic: 144.5 (13.4)/138.5 (4.3)	Garlic: 91 (3.9)/87 (3.7)
	Garlic/placebo	900 mg/d,		Control: 144 (10.4)/147 (7.1)	Control: 88 (6.1)/90 (3.7)
		16 wks			
Holzgartner et al. 1992, [14]	Parallel,	Kwai,	47/47	Garlic: 143.4 (15.4)/135.4 (14.6)	Garlic: 82.8 (10.5)/78.6 (9.3)
	Lipid-lowering drug (Benzafibrate) + garlic/drug only	900 mg/d,		Control: 140.6 (18.7)/137.2 (14.6)	Control: 82.4 (9.5)/78.4 (9.2)
		12 wks			
Kiesewetter et al. 1993, [15]	Parallel,	Kwai,	32/32	Not reported	Garlic: 84.7 (13.7)/81.7 (12.1)
	Garlic/placebo	800 mg/d,			Control: 83.3 (11)/81.7 (11)
		12 wks,			
Jain et al. 1993, [16]	Parallel,	Kwai,	20/22	Garlic: 129 (13)/130 (17)	Garlic: 82 (6)/81 (10)
	Garlic/placebo	900 mg/d,		Control: 128 (10)/127 (12)	Control: 83 (8)/82 (6)
		12 wks			
Saradeth et al. 1994, [17]	Parallel,	Kwai,	25/27	Garlic: 125 (17)/127.4 (16)	Garlic: 80.8 (8)/82.7 (10)
	Garlic/placebo	600 mg/d,		Control: 124.6 (15.6)/122.8 (12.5)	Control: 81.8 (9.4)/81.1 (9.4)
		15 wks			
Simons et al. 1995, [18]	Crossover,	Kwai,	28/28	Garlic: 127 (14)/119 (7)	Garlic: 80 (8)/76 (5)
	Garlic/placebo	900 mg/d,		Control: 127 (14)/122 (10)	Control: 80 (8)/76 (6)
		12 wks			
Steiner et al. 1996, [19]	Parallel study arm,	Aged garlic extract,	41/41	Garlic: 134 (14)/126 (14)	Garlic: 84 (8.6)/82.3 (9)
	Garlic/placebo	2400 mg/d,		Control: 134 (11)/129.6 (12)	Control: 85 (7.4)/81.7 (8)
		23 wks			
Adler & Holub 1997, [20]	Parallel,	Kwai,	12/13/10/11	Garlic: 123.3 (14.5)/118.5 (9.4)	Garlic: 83.2 (2.5)/80 (2.2)
	Garlic/garlic+fish oil/fish oil/placebo	900 mg/d,		Control: 118.3 (3.2)/119.6 (3)	Control: 79.6 (2.2)/80.9 (2)
		12 wks			
Zhang et al. 2000, [21]	Parallel,	Distilled garlic oil,	14/13	Garlic: 117 (8)/113.5 (6.4)	Garlic: 72 (7)/68.2 (9.4)
	Garlic/placebo	12.3 mg/d,		Control: 109 (9)/109.9 (9.4)	Control: 64 (7)/62.8 (5.4)
		16 wks			

SBP, systolic blood pressure; DBP, diastolic blood pressure; SD, standard deviation; vs, versus; mm Hg, millimetre mercury; mg/d, milligram per day; wks, weeks; mths, months.

### Data extraction and quality assessment for meta-analysis

The number of subjects in intervention and control groups, mean SBP and DBP at start and end of intervention and SD were collated from text, tables or figures. Methodological quality was assessed independently by two investigators (KR and PF) using guidelines by the Cochrane Collaboration [44] (Tables 2 and 3), and disagreements were resolved by consensus. All studies considered for meta-analysis (n = 11, Table 2) reported adequate randomisation and double blinding, and all but one [14] assessed blood pressure as co-primary outcome measure. Six of eleven studies reported drop-out rates between 0 and 13% [13,14,17,18,20,21], two studies of less than 22% [15,19], and three studies did not provide details [11,12,16]. We considered the quality of all eleven studies as sufficient to be included in meta-analysis.

**Table 2:** Assessment of study quality for studies included in meta-analysis

**Study ID**	**Randomisation**	**Blinding**	**Outcome measure: Blood pressure**	**Loss of follow up**	**Funding source**
Kandziora J 1988 (Study 1), [11]	+	++	Primary, Mean of 2 readings each standing + supine	Unclear	-
Auer et al. 1990, [12]	+	++	Primary, Mean standing + supine	Unclear	-
Vorberg & Schneider 1990, [13]	+	++	Primary, Mean standing + supine	G: 0%, C: 0%	-
Holzgartner et al. 1992, [14]	+	++	Secondary, Unclear	G: 4.8%; C: 4.8%; T: 4.8%	-
Kiesewetter et al. 1993, [15]	+	++	Primary, Riva Rocci method	G: 20%; C: 20%; T: 20%	-
Jain et al. 1993, [16]	+	++	Primary, Mean of 2 readings after 10 min rest; standard technique (JNC 1988)	Unclear	Industry grant
Saradeth et al. 1994, [17]	+	++	Primary, Riva Rocci method	G: 2.8%; C: 8.3%; T: 5.6%	-
Simons et al. 1995, [18]	+	++	Primary, Mean of 2 readings after 5 min rest, phase V diastolic BP	T: 9.7%	Industry grant
Steiner et al. 1996, [19] parallel arm	+	++	Primary, Unclear, manual	T: 21.2%	-
Adler & Holub 1997, [20]	+	++	Primary, Sitting digital	T: 8%	Heart & Stroke Foundation
Zhang et al. 2000, [21]	+	++	Primary, Over 10–30 min until repeated low values were obtained, means of 3 lowest pulse rates + associated BP values	G: 6.7%; C: 13.3%; T: 10%	Industry grant

+: adequate; ++: double blinding; -: not provided

**Table 3:** Assessment of study quality for studies excluded from meta-analysis

**Study ID**	**Randomisation**	**Blinding**	**Outcome measure: Blood pressure**	**Loss of follow up**	**Funding source**
Lutomski 1984, [22]	+	++	Primary, Unclear	G: 13.7%; C: 25.5%; T: 20.4%	-
Barrie et al. 1987, [23]	+	++	Primary, Mean bilateral	Unclear	Industry grant
Harenberg et al. 1988, [24]	No (simple intervention)	Open label	Primary, Unclear	None	-
Kandziora J. 1988 (Study 2), [25]	+	+ Observer blinded	Primary, Mean of 2 readings each standing + supine	Unclear	-
Kiesewetter et al. 1991, [26]	Unclear	++	Unclear	Unclear	-
DeASantos & Gruenwald 1993, [27]	+	++	Primary, Unclear	G: 16.7%; C: 10%; T: 13.3%	Industry grant
DeASantos & Johns 1995, [28]	+	Open label	Primary, Average of 3 readings	G: 10%; C: 15%; T: 12.5%	-
Czerny & Samochowiek 1996, [29]	+	++	Primary, Unclear (after 15 min exercise)	Unclear	-
Mansell et al. 1996, [30]	+	Unclear	Primary, Unclear	Unclear	-
Steiner et al. 1996, [19] crossover arm	+	++	Primary, Unclear, manual	T: 21.2%	-
McCrindle et al. 1998, [31]	+	++	Primary, Unclear	No drop-outs	-
Durak et al. 2004, [32]	No (hypertensive/nomotensive)	Open label	Unclear	Unclear	-
Turner et al. 2004, [33]	+	++	Secondary, Mean of 2 readings after 10 min rest	G: 6.1%; C: 5.9%; T: 6.0%	Industry grant
Dhawan & Jain 2004, [34]	Unclear (hypertensives/normotensives)	++	Primary, As per JNC VI recommendations 2× after 10 min rest DBP determined as Korotkoff phase V	No drop-outs	Council of Medical Research grant
Jabbari et al. 2005, [35]	+	Open	Primary, Unclear	G: 12%; C: 12%; T: 12%	-

+: adequate; ++: double blinding; -: not provided; JNC: Joint National Committee

### Data synthesis and meta-analysis

Changes in mean SBP or DBP of garlic and control groups before and after intervention were entered into the meta-analysis using Review Manager version 4.2 [45]. Standard deviations of these differences were estimated applying the equation published in Taubert et al. [46] and using a conservative correlation coefficient of R = 0.68 as suggested by the Cochrane Handbook for Systematic Reviews of Interventions [44]. If heterogeneity was high (I^2^>50%) we used the random effects model for meta-analysis, otherwise the fixed effects model was considered appropriate [47,48].

In addition, we performed subgroup meta-analysis of trials with hypertensive subjects at start of treatment (mean SBP ≥ 140 mm Hg or mean DBP ≥ 90 mm Hg) and subgroup analysis of trials with normotensive subjects at start of treatment (mean SBp < 140 mm Hg or mean DBp < 90 mm Hg).

Potential publication bias in the meta-analysis was assessed by Begg's funnel plots and Egger's regression test [49,50].

Meta-regression was performed to find any association between blood pressure changes over time and the following continuous variables: dosage, length of intervention, and blood pressure at start of treatment. We also tested for any evidence of confounding related to source of funding (details on funding in Tables 2 and 3). Regression analyses were conducted using Stata version 9 [51].

### Plotting of blood pressure changes over time

We integrated additional blood pressure data from studies included in the systematic review in BP/time plots for visual assessment of BP trends depending on BP at start of treatment (mean/median SBP at start ≥ or < 130 mm Hg, mean/median DBP ≥ or < 85 mm Hg). We included in the plots data of placebo and non-placebo controlled trials using garlic-only preparations, and reporting mean or median SBP and/or DBP.

## Results

Eleven of 25 studies included in our systematic review and investigating the effect of garlic on blood pressure met the inclusion criteria for meta-analysis (Table 1) [11-21]. Fourteen studies were excluded from meta-analysis: six had no placebo control group [24,25,28,32,34,35], another six reported incomplete data for mean SBP, DBP or SD [23,26,27,30,31,33], and another two because they used garlic combination supplements containing other potentially hypotensive agents [22,29] (Additional File 1). We were able to contact the authors of four studies with suitable study design but incompletely reported data required for meta-analysis [20,26,27,33], and obtained complete data from one study [20].

Ten of the eleven studies included in the meta-analysis reported complete SBP and SD data required for meta-analysis, and eleven studies reported DBP and SD data (Table 1) (DBP data only [15]). Nine studies compared garlic preparations to placebo, and two studies compared the effect of garlic on blood pressure in addition to a drug compared to drug plus placebo [11: diuretic, antihypertensive, acts on sodium chloride reabsorption, 14: lipid-lowering drug]. Nine studies used garlic powder (mainly "Kwai", a standardised garlic supplement [52]), one study used aged garlic extract [19] and another used distilled garlic oil [21]. Dosage of garlic powder ranged between 600 and 900 mg per day, and duration of intervention ranged from 12 to 23 weeks. A total of 252 individuals allocated to a garlic intervention group and 251 individuals allocated to a control group were included in the meta-analysis on SBP, and 283 (garlic) versus 282 (control) on DBP. Mean blood pressure at start of intervention varied markedly, with four studies reporting mean SBP in the hypertensive range (≥140 mm Hg) and three studies reporting mean DBP in the hypertensive range ≥90 mm Hg) before treatment.

**Figure 2:**
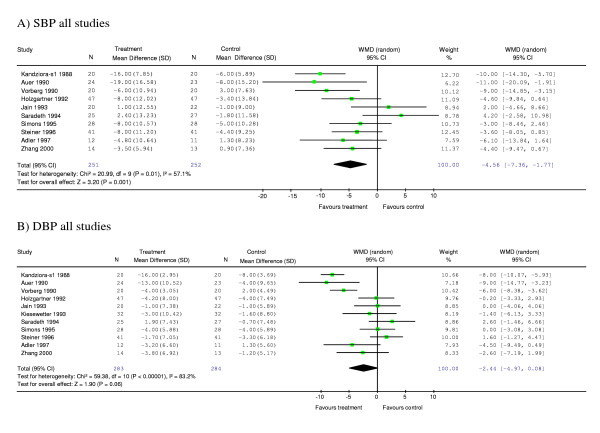
**Meta-analysis graphs on the effect of garlic on systolic blood pressure (A) or diastolic blood pressure (B)**. *Abbreviations: N, number of participants; SD, standard deviation; WMD, weighted mean difference; CI, confidence interval; SBP, systolic blood pressure; DBP, diastolic blood pressure; s1, study 1 [ref 11]*.

**Figure 3:**
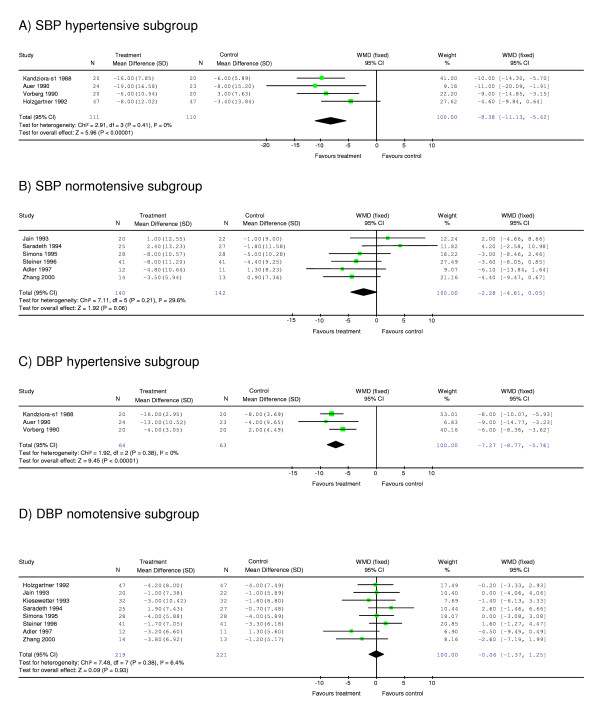
**Subgroup meta-analysis on the effect of garlic on systolic blood pressure of hypertensive subjects (≥140 mm Hg at start of intervention) (A) or 'normotensive' subjects (<140 mm Hg at start of intervention) (B); on diastolic blood pressure of hypertensive subjects (≥90 mm Hg) (C) or normotensive subjects (<90 mm Hg) (D)**. *For abbreviations see Fig 2*.

Meta-analysis of ten studies of the effect of garlic on SBP showed a significant difference between garlic and control groups, with garlic having a greater effect in reducing SBP than placebo by 4.56 [95% CI (confidence interval), -7.36, -1.77] mm Hg compared with placebo (p < 0.001) (Figure 2a). Subgroup analysis of studies with mean SBP in the hypertensive range at start of intervention revealed a greater SBP reduction in the garlic group than placebo by 8.38 [95% CI, -11.13, -5.62] mm Hg (p < 0.001) (Figure 3a). Subgroup analysis of the remaining studies with mean SBP in the normotensive range (<140 mm Hg) at start of intervention showed no significant difference between the garlic and placebo groups (Figure 3b).

Meta-analysis of eleven studies of the effect of garlic on DBP did not show a significant difference between garlic and placebo groups (-2.44 [95% CI, -4.97, 0.09] mm Hg, p = 0.06) (Figure 2b). However, subgroup analysis of studies with mean DBP in the hypertensive range at the start of treatment revealed a significant difference between garlic and control groups. The results indicate that garlic was more effective in reducing DBP than placebo in hypertensive individuals by 7.27 [95% CI, -8.77, -5.76] mm Hg (p < 0.001) (Figure 3c). In contrast, subgroup meta-analysis of "normotensive" individuals was not significant (Figure 3d).

Heterogeneity was moderate for meta-analysis of SBP of all ten studies (I^2 ^= 57.1%). However, we found no heterogeneity in the subgroup analysis of studies with hypertensive individuals at start of intervention (I^2 ^= 0%). The same trend was observed for meta-analysis of DBP with I^2 ^= 83.2% for pooled analysis of all ten studies and I^2 ^= 0% for subgroup analysis of studies with hypertensive subjects at start of intervention.

Regression analysis was conducted to test whether heterogeneity between the studies could be explained by one or more of the following continuous variables: dosage (only studies using garlic powder were included, n = 8/9 (SBP/DBP), range 600–900 mg/d), duration of intervention (SBP/DBP: n = 10/11, range 12–23 wks), and SBP or DBP at start of intervention (SBP/DBP: n = 10/11, range 175–109 SBP/102-64 DBP). SBP or DBP at start of intervention proved to be a significant predictor for heterogeneity (SBP: R = -0.151, p = 0.03; DBP: R = -0.316, p = 0.02), strengthening the results of subgroup meta-analysis. None of the other variables tested showed a significant association with blood pressure outcomes (data not shown). Furthermore, regression analysis did not provide any evidence to suggest that receipt of industry funding (n = 3) was associated with blood pressure outcomes.

**Figure 4:**
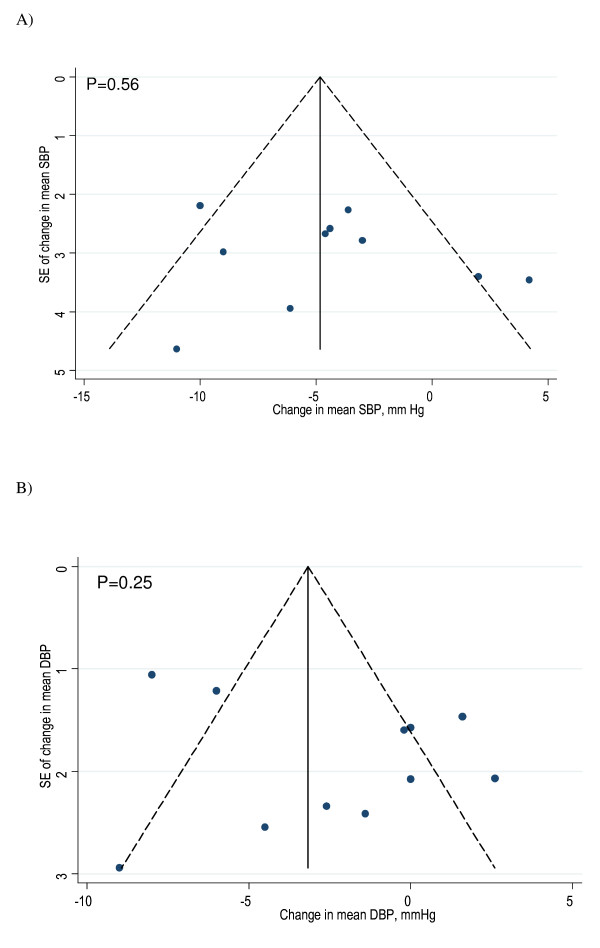
**Funnel plots of studies included in meta-analysis on the effect of garlic on systolic blood pressure (A) and diastolic blood pressure (B)**. The vertical line of Begg's funnel plot represents the pooled mean effect size, the dotted lines the 95% confidence interval, p-values are derived from Egger's test. *Abbreviations: SB, systolic blood pressure; DBP, diastolic blood pressure; SE, standard error; mm Hg, millimetre mercury*.

Funnel plots and Egger regression tests suggested no publication bias in the meta-analyses (Figure 4).

**Figure 5:**
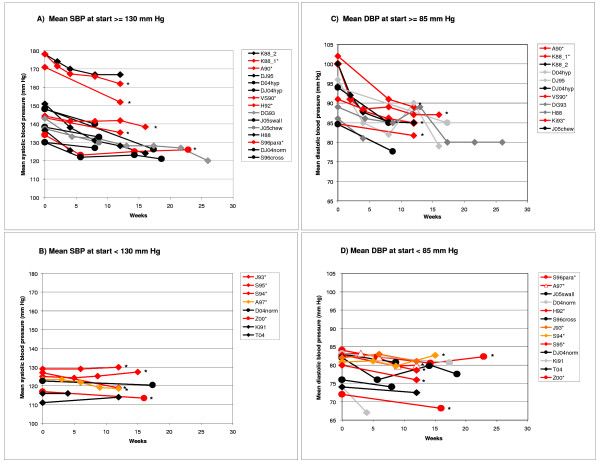
**Mean BP against time for garlic-only intervention arm(s) of studies using subjects with SBP≥130 mm Hg at start of intervention (A), SBP < 130 mm Hg (B), DBP≥85 mm Hg (C), DBP < 85 mm Hg (D)**. The plot incorporates garlic-only intervention arms of studies included in the systematic review: study arms in meta-analysis are in red/orange and marked with *, others are in black/grey. Diamonds illustrate trials using garlic powder and circles illustrate other garlic preparations. Studies in legend boxes are sorted by baseline blood pressure. Abbreviations: K88_2 = Kandziora 1988 (Study 2) [25], garlic vs drug; K88_1*= Kandziora 1988 (Study 1) [11], garlic+drug vs placebo+drug; A90* = Auer et al. 1990 [12]; DJ95 = De A Santos & Johns 1995 [28]; D04hyp = Durak et al. 2004 [32], hypertensive study arm; DJ04hyp = Dhawan & Jain 2004 [37], hypertensive study arm; VS90* = Vorberg & Schneider 1990 [13]; H92*= Holzgartner et al. 1992 [15]; DG93 = De A Santos & Grünwald 1993 [27]; J05swall = Jabbari et al. 2005 [35], swallowing garlic study arm; J05chew = Jabbari et al. 2005 [35], chewing garlic study arm; H88 = Harenberg et al. 1988 [24]; S96para = Steiner et al. 1996 [19], parallel study arm ; DJ04norm = Dhawan & Jain 2004 [34], normotensive study arm; S96cross = Steiner et al. 1996 [19], crossover study arm; J93 = Jain et al. 1993 [16]; S95 = Simons et al.1995 [18]; S94 = Saradeth et al. 1994 [17]; D04norm = Durak et al. 2004 [32], normotensive study arm ; Z00 = Zhang et al. 2000 [21]; Ki91 = Kiesewetter 1991 [26]; T04 = Turner et al. 2004 [33], median BP.

Figure 5 augments our systematic review, allowing a visual comparison of BP changes in garlic-only intervention arms of published randomised trials (Table 1 and Additional File 1). Blood pressure changes over time were plotted by blood pressure at start of treatment using data from 20 out of the 25 studies identified in our systematic review, which included placebo and non-placebo controlled trials, using garlic-only preparations, and reporting mean or median SBP and/or DBP (studies in meta-analysis [11-21]; as well as garlic-only intervention arms included in systematic review [24-28,32,33,35,53]). In total, mean or median SBP data was available for 23 garlic-only intervention arms and mean or median DBP data for 24 garlic intervention arms. Studies of groups with high blood pressure (mean SBP or DBP) at start of intervention generally showed a downward trend of blood pressure over time (Figure 5a and 5c). These included studies of groups with high normal blood pressure, also known as pre-hypertension (SBP≥130 mm Hg, DBP≥85 mm Hg). Blood pressure generally changed little for studies of groups with mean SBP lower than 130 mm Hg or mean DBP lower than 85 mm Hg at start of intervention (Figure 5b and 5d).

## Discussion

Our meta-analysis suggests that garlic supplementation exerts a hypotensive effect compared to placebo, in particular in individuals with high blood pressure (SBP ≥ 140 mm Hg, DBP ≥ 90 mm Hg). Meta-analysis of all studies showed a mean decrease of 4.6 ± 2.8 mm Hg for SBP in the garlic group compared to placebo (p = 0.001), while the mean decrease in the hypertensive subgroup was 8.4 ± 2.8 mm Hg for SBP and 7.3 ± 1.5 mm Hg for DBP (p < 0.00001). Low heterogeneity in the subgroup analyses in addition to regression analysis confirmed that starting blood pressure was a significant predictor for treatment effect of garlic on blood pressure.

Interestingly, garlic-only intervention arms of reviewed studies which were not suitable for meta-analysis also showed a trend for greater reduction in BP with higher starting BP. These observed trends are in line with the findings from our subgroup meta-analyses, supporting the evidence for a hypotensive effect of garlic in individuals with high or high normal blood pressure.

Whilst data from two studies [26,27], included in a previous meta-analysis [41], were not available, our meta-analysis incorporates data from six additional recent studies [15,17-21], allowing subgroup analysis and increasing generalisability. Quality of studies included in the meta-analysis was generally high, however, 20% loss to follow up in two trials and non-reporting of drop out rates in three trials might have biased the results in those studies.

Heterogeneity observed in the meta-analyses including all studies could only partly be explained by starting BP, while dosage and duration of treatment were not associated with BP outcome. The absence of an association between dosage and blood pressure change may suggest that the hypotensive effect of garlic is comparable for dosages between 600 and 900 mg per day of Kwai powder. On the other hand, detection of an association between duration of garlic intake and blood pressure change may have been limited because the majority of studies (7 out of 11) took final BP measurements at 12 weeks.

Our findings of the effect of garlic preparations on SBP/DBP are comparable to the hypotensive effects of commonly-prescribed blood pressure drugs, e.g. beta-blockers of 5 mm Hg for SBP, angiotension converting enzyme inhibitors (ACEI) of 8 mm Hg for SBP [54], and angiotensin II type 1 receptor antagonists of 10.3 mm Hg for DBP [55]. Our findings may have implications at a population level, where a reduction of 4 to 5 mm Hg in SBP and 2 to 3 mm Hg in DBP has been estimated to reduce the risk of cardiovascular morbidity and mortality by 8–20% [56]. While our study focuses on the short-term effects of garlic on blood pressure, larger scale long-term trials are needed to test the effectiveness of garlic on cardiovascular outcomes.

Most studies included in this review used garlic powder dosages of 600–900 mg per day, providing potentially 3.6–5.4 mg of allicin, the active compound in garlic [36]. In comparison, fresh garlic cloves (~2 g) each yield 5–9 mg allicin [2]. However, different garlic preparations have variable effectiveness on blood pressure, e.g. minimal allicin compounds are found in aged garlic extract or heat treated garlic, which may limit its hypotensive properties [4,5]. Therefore it is advisable to use standardised garlic preparations in future trials [57,58].

Supplementation with garlic preparations compared to raw garlic provides the advantage of reducing or avoiding garlic breath and body odour, and prevents possible destruction of active compounds in the cooking process. Since garlic generally has a high tolerability [36], supplementation with garlic preparations may provide an acceptable alternative or complementary treatment option for hypertension.

Future research investigating a dose-response relationship between standardised garlic preparations and blood pressure would be warranted, as limited data are available [59]. Moreover, future trials could investigate whether garlic has a blood pressure reducing effect in pre-hypertensive subjects (SBP 120–139 mmHg, DBP 80–90 mm Hg), which may help to forestall progression to hypertension [1,60].

## Conclusion

This systematic review and meta-analysis suggests that garlic preparations are superior to placebo in reducing blood pressure in individuals with hypertension. Future large scale long-term trials are needed to investigate whether standardised garlic preparations could provide a safe alternative or complementary treatment option for hypertension in clinical practice.

## List of Abbreviations

BP: blood pressure; CI: confidence interval; DBP: diastolic blood pressure; mg/d: milligram per day; mm Hg: millimetre mercury; RCT: randomised controlled trial; SBP: systolic blood pressure; SD: standard deviation.

## Competing interests

The authors declare that they have no competing interests.

## Authors' contributions

KR, ORF and NPS conceptualised the study and obtained funding. Data was acquired independently by KR and PF. KR, PF and TS undertook data analysis and interpretation. KR prepared the manuscript with contributions from all co-authors. All authors approved the final version.

## Pre-publication history

The pre-publication history for this paper can be accessed here:



## Supplementary Material

Additional file 1 Characteristics of studies excluded from the meta-analysis examining the effect of garlic on blood pressure. Click here for file
