# Supplementary material for: Effect of garlic on blood pressure: A systematic review and meta-analysis
Source: BMC Cardiovasc Disord. 2008 Jun 16;8:13. doi: 10.1186/1471-2261-8-13 (PMC2442048; doi:10.1186/1471-2261-8-13)
Supplement: Additional file 1 — Characteristics of studies excluded from the meta-analysis examining the effect of garlic on blood pressure. [file 1471-2261-8-13-S1.pdf]

Additional file 1: Characteristics of studies excluded from the meta-analysis examining the effect of garlic on blood pressure

Source	Study design, Intervention/ control groups	Type of garlic preparation, Dosage, Duration	Number of participants in intervention vs control group	Main outcomes, SBP/DBP (SD) in intervention groups	Reason for exclusion in meta-analysis
Lutomski 1984, [22]	Parallel,	Garlic combination preparation (Ilja Rogoff pills),	44/38	Garlic: 77% SBP: reduction of ≥ 20mm Hg with start SBP ≥ 150 mm Hg; DBP: reduction of ≥ 10mm Hg with start DBP ≥ 100 mm Hg	Garlic combination prep. used; no mean SBP/DBP reported
	Garlic combination/placebo	300 mg/d,		Control: 57% reduction of SBP/DBP	
		12 wks			
Barrie et al. 1987, [23]	Crossover,	Cold-pressed garlic oil,	20/20	Garlic: mean BP 94.2 (22 SEM)/88.7 (14 SEM),	Only mean BP data given, not SBP/DBP
	Garlic/placebo	18 mg/d (extracted from 9g fresh garlic),		Control: mean BP 96.9 (12 SEM)/94.6 (15 SEM)	
		4 wks			
Harenberg et al. 1988, [24]	Simple intervention study,	Dried garlic,	20	Garlic: SBP 136.5/125.5, DBP 86/81	No control group, no SD reported
	Garlic group only	600 mg/d,			
		4 wks			
Kandziora J. 1988	Parallel,	Kwai,	20/20	Garlic: SBP 176 (6)/164 (7), DBP 99 (1)/85 (2)	No true placebo group

(Study 2) , [25]					
	Garlic/diuretic drug (Reserpin)	600 mg/d,		Drug group: SBP 175 (4)/ 162 (5), DBP 98 (1)/ 84 (3)	
		12 wks			
Kiesewetter et al. 1991, [26]	Parallel,	Dried garlic powder,	30/30	Garlic: SBP 116 (11)/no change, DBP 74 (9)/67 (5)	Incomplete data set at end of intervention (e.g. mean SBP/DBP control group)
	Garlic/placebo	800 mg/d,		Control: SBP 116 (11)/no change, DBP 73 (8)/not reported	
		4 wks			
DeASantos & Gruenwald 1993, [27]	Parallel,	Kwai,	25/27	Garlic: SBP 143 (21) /120, DBP 89 (11)/80	No SD at end of intervention reported
	Garlic/placebo	900 mg/d,		Control: SBP 144 (17)/144, DBP 89 (11)/89	
		26 wks (6 mths)			
DeASantos & Johns 1995, [28]	Parallel,	Kwai / garlic oil capsules,	36/34	Garlic powder: SBP 151 (24)/124 (12), DBP 96 (12)/79 (6)	No true placebo group, comparison of different garlic preparations
	Garlic powder/ garlic oil	600 mg/d / 1.98 mg/d,		Garlic oil: SBP 138 (17.5)/138 (17.5), DBP 88 (5.8)/86 (11.7)	
		17.5 wks (4 mths)			
Czerny &	Parallel,	Garlic oil + hawthorn +	50/50	Garlic: SBP 163/144, DBP 100/93	Garlic combination

Samochowiek 1996, [29]		lecithin + wheat germ			preparation used, only graphs, no SD
	Garlic combination/placebo	400 mg/d		Control: SBP 164/155, DBP 104/94	
		17.5 wks (4 mths)			
Mansell et al. 1996, [30]	Parallel,	Kwai,	30/30	“No significant effect on BP.”	No BP data reported
	Garlic/placebo	900 mg/d,			
		12 wks			
Steiner et al. 1996, [19]	Crossover study arm,	Aged garlic,	41/41	Garlic: SBP 130 (13)/121 (12), DBP 82 (8)/78 (8)	Data of parallel study arm of same individuals included in meta-analysis
	Garlic/placebo	2400 mg/d,		Control: SBP 130 (16)/128 (11), 81 (9)/80 (6)	
		23 wks			
McCrindle et al. 1998, [31]	Garlic/placebo	Kwai,	15/15,	Garlic: SBP 102 (9)/ +2.1 (-7.1, +11.3) absolute effect; +2.3 (-6.8, +11.4) relative effect , DBP 63 (10)/ 0 (-6.5, +6.5) absolute effect; -0.4 (-11.9, +11.1) relative effect	No Mean SBP/DBP + SD given at end of intervention, only absolute and relative effect + confidence interval
		900 mg/d,	Children (8-18 years)	Control: SBP 102 (9)/?, DBP 60 (8)/?	
		8 wks			
Durak et al. 2004, [32]	Parallel,	Aqueous garlic extract (20% w/v),	13 hypertensive/ 10 normotensive	Garlic group 1: SBP 148.3 (29.2)/126.2 (15.6), DBP 98.5 (22.3)/85.1 (12.4)	No placebo group

	Garlic/garlic	~ 10 g/d,		Garlic group 2:SBP 122.6 (15.4)/120.4 (12.8), DBP 83.2 (13.2)/80.6 (10.8)	
		17.5 wks (4 mths)			
Turner et al. 2004, [33]	Parallel,	Garlic powder tablets,	29/30	Garlic: median SBP 111 (106-121.8)/ median 114(106.5-123.3), median DBP 73.5 (68.3-78.5)/72.5 (69.5-81.8)	No mean SBP/DBP (SD) at end of treatment, only median + interquartile range reported
	Garlic/placebo	Approx. 500 mg /d,		Control: median SBP 115 (104.9-128.5)/119 (109-131), median DBP 74.5 (69.9-80.6)/77 (71.9-82.9)	
		12 wks			
Dhawan & Jain 2004, [34]	Parallel,	Garlic pearls,	20 hypertensives/20 normotensives	Garlic group 1: SBP 148 (12)/ 140 (16), DBP 94 (15)/85 (23)	No placebo group
	Garlic/Garlic	250 mg/d,		Garlic group 2: SBP 130 (22)/127 (17), DBP 76 (12)/74 (20)	
		8 wks			
Jabbari et al. 2005, [35]	Crossover,	Raw aged garlic,	25/25	Chewing garlic group: SBP 137.5 (21.7) /129.8 (25.5), DBP 84.6 (9.5)/77.6 (9.7)	No placebo group, not stated if SD or SEM given.
	Chewing/swallowing	1 clove/d,		Swallowing garlic group: SBP 138.2 (23.6)/132.8 (23.4), DBP 83 (10.5)/80.7 (9.7)	
		8 wks (2 mths)			

SEM = standard error, see table 1 for other abbreviations
